# 1-Ferrocenyl-3-(2-methyl­anilino)propan-1-one

**DOI:** 10.1107/S1600536812028802

**Published:** 2012-06-30

**Authors:** Zorica Leka, Sladjana B. Novaković, Anka Pejović, Goran A. Bogdanović, Rastko D. Vukićević

**Affiliations:** a Faculty of Metallurgy and Technology, University of Montenegro, Cetinjski put bb, 81000 Podgorica, Montenegro; b ’Vinča’ Institute of Nuclear Sciences, Laboratory of Theoretical Physics and Condensed Matter Physics, PO Box 522, University of Belgrade, 11001 Belgrade, Serbia; c Department of Chemistry, Faculty of Science, University of Kragujevac, R. Domanovića 12, 34000 Kragujevac, Serbia

## Abstract

In the ferrocene-containing Mannich base, [Fe(C_5_H_5_)(C_15_H_16_NO)], the dihedral angle between the mean planes of the benzene ring and the substituted cyclo­penta­dienyl ring is 84.63 (7)°. The conformation of the title compound significantly differs from those found in corresponding *m*-tolyl­amino and *p*-tolyl­amino derivatives. In the crystal, C—H⋯O inter­actions connect the mol­ecules into chains, which further inter­act by means of C—H⋯π inter­actions. It is noteworthy that the amino H atom is shielded and is not involved in hydrogen bonding.

## Related literature

For the physico-chemical properties of ferrocene-based compounds see: Togni & Hayashi (1995 ▶). For related structures and details of the synthesis, see: Damljanović *et al.* (2011 ▶); Pejović *et al.* (2012 ▶); Stevanović *et al.* (2012 ▶); Leka *et al.* (2012*a* ▶,*b* ▶,*c* ▶).
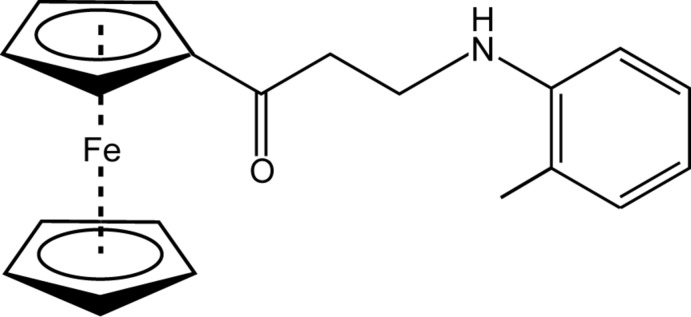


## Experimental

### 

#### Crystal data

[Fe(C_5_H_5_)(C_15_H_16_NO)]
*M* *_r_* = 347.23
Monoclinic, 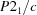

*a* = 12.1343 (4) Å
*b* = 17.8010 (7) Å
*c* = 7.5464 (2) Å
β = 92.946 (3)°
*V* = 1627.89 (9) Å^3^
*Z* = 4
Mo *K*α radiation
μ = 0.93 mm^−1^
*T* = 293 K
0.22 × 0.18 × 0.12 mm

#### Data collection

Oxford Diffraction Xcalibur Sapphire3 Gemini diffractometer
Absorption correction: multi-scan (*CrysAlis PRO*; Oxford Diffraction, 2009 ▶)’ *T*_min_ = 0.923, *T*_max_ = 1.000
7605 measured reflections
3694 independent reflections
2843 reflections with *I* > 2σ(*I*)
*R*_int_ = 0.029

#### Refinement

*R*[*F* ^2^ > 2σ(*F* ^2^)] = 0.039
*wR*(*F* ^2^) = 0.097
*S* = 1.04
3694 reflections
213 parameters
H atoms treated by a mixture of independent and constrained refinement
Δρ_max_ = 0.28 e Å^−3^
Δρ_min_ = −0.28 e Å^−3^

### 

Data collection: *CrysAlis PRO* (Oxford Diffraction, 2009 ▶); cell refinement: *CrysAlis PRO*; data reduction: *CrysAlis PRO*; program(s) used to solve structure: *SHELXS97* (Sheldrick, 2008 ▶); program(s) used to refine structure: *SHELXL97* (Sheldrick, 2008 ▶); molecular graphics: *ORTEP-3* (Farrugia, 1997 ▶) and *Mercury* (Macrae *et al.*, 2006 ▶); software used to prepare material for publication: *WinGX* (Farrugia, 1999 ▶), *PLATON* (Spek, 2009 ▶) and *PARST* (Nardelli, 1995 ▶).

## Supplementary Material

Crystal structure: contains datablock(s) I, global. DOI: 10.1107/S1600536812028802/bt5950sup1.cif

Structure factors: contains datablock(s) I. DOI: 10.1107/S1600536812028802/bt5950Isup2.hkl

Additional supplementary materials:  crystallographic information; 3D view; checkCIF report

## Figures and Tables

**Table 1:** Hydrogen-bond geometry (Å, °) *Cg* is the centroid of the C14–C19 ring.

*D*—H⋯*A*	*D*—H	H⋯*A*	*D*⋯*A*	*D*—H⋯*A*
C12—H12*A*⋯O1^i^	0.97	2.38	3.182 (3)	139
C19—H19⋯*Cg*1^i^	0.93	2.98	3.838 (3)	160

Symmetry code: (i) 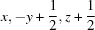.

**Fig. 1.**
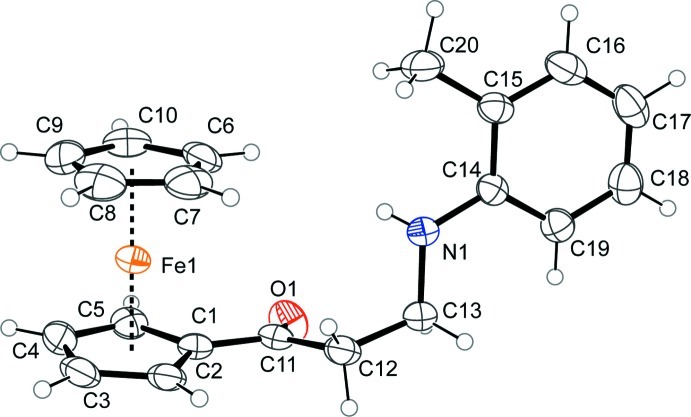
The molecular structure of (I), with atom labels and 40% probability displacement ellipsoids for non-H atoms.

**Fig. 2.**
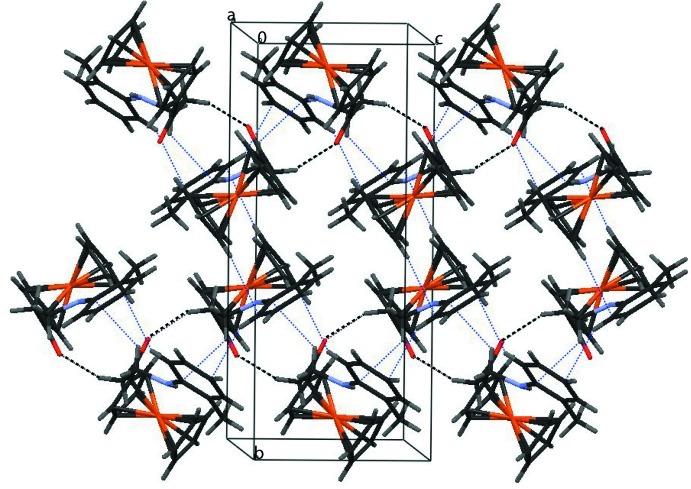
Segment of the crystal packing. The C12—H···O1 interactions connecting the molecules into chains are indicated by black dotted lines. C—H···π interactions are given in blue doted lines.

## supplementary crystallographic information

### Comment

The title compound 1-Ferrocenyl-3-(*o*-tolylamino)propan-1-one (I), Fig. 1, shows considerable conformational differences in comparison to the crystal structures of two closely related derivatives, 1-Ferrocenyl-4-(*m*-tolylamino)propan-1-one (Pejović *et al.*, 2012) and 1-Ferrocenyl-3-(*p*-tolylamino)propan-1-one (Leka *et al.*, 2012*b*). The torsion angles C1—C11—C12—C13, C11—C12—C13—N1 and C12—C13—N1—C4 within the aliphatic fragment have the values of -161.7 (2), 78.9 (3) and 168.9 (2)°. The latter torsion angle which defines the final orientation of the phenyl ring significantly differs from the values found in *m*-tolylamino [69.4 (4)°] and *p*-tolylamino [70.6 (3)°] derivatives. On the other hand, the conformation of the title compound is closer to the one found in those 3-(arylamino)-1-ferrocenylpropan-1-ones which comprise other *ortho* substituted arylamino fragments, such as previously reported 1-Ferrocenyl-3-(2-acetylphenylamino)propan-1-one (Stevanović *et al.*, 2012) and 1-Ferrocenyl-3-(2-nitrophenylamino)propan-1-one (Damljanović *et al.*, 2011), [the torsion angle C12—C13—N1—C4 in these compounds has the value -176.1 (6) and -175.7 (6)° respectively]. In the molecule of (I) the phenyl ring is nearly orthogonally positioned with regard to substituted Cp ring. The dihedral angle between the mean planes of the phenyl ring and the substituted Cp ring is 84.63 (7)°. The Cp rings within the Fc unit display nearly eclipsed conformation with C1—Cg1—Cg2—C6 angle of 9.93° (Cg is centroid of the corresponding Cp ring). The molecules of (I) connect *via* C12–H12*a*···O1 interaction into zigzag chain extended along *c* axis (Fig. 2). The chains are further related by means of extensive C—H···π interactions, C19—H19···Cg1^i^: H···Cg 2.98 Å, H-Perp 2.87 Å, *X*—H···Cg 160°, (i = *x*, -*y* + 1/2, *z* - 1/2); C8—H8···Cg1^ii^: H···Cg 3.02 Å, H-Perp 2.84 Å, *X*—H···Cg 140° (ii = -*x* + 1, -*y*, -*z* + 1); C13—H13*b*···Cg1^i^: H···Cg 3.35 Å, H-Perp 2.87 Å, *X*—H···Cg 127°; C16—H16···Cg2^iii^: H···*Cg* 3.07 Å, H-Perp 2.97 Å, *X*—H···*Cg* 168 ° (iii = -*x* + 1, -*y*, -*z* + 1); C20—H20*a*···Cg2^iii^: H···Cg 3.38 Å, H-Perp 2.95 Å, *X*—H···Cg 140° (Cg1 and Cg2 are centroids of phenyl and unsubstituted Cp ring respectively).

### Experimental

The compound was obtained by an aza-Michael addition of the coresponding arylamine to acryloylferrocene. The reaction was performed by microwave (MW) irradiation (500 W/5 min) of a mixture of reactants and montmorillonite K-10, without a solvent as described by Damljanović *et al.* (2011).

### Refinement

H atoms bonded to C atoms were placed at geometrically calculated positions and refined using a riding model. C—H distances were fixed to 0.93, 0.97 and 0.96 Å from aromatic, methylene and methyl C atoms, respectively. The *U*_iso_(H) values set to 1.2 times *U*_eq_ of the corresponding C atoms (1.5 for methyl groups). The H atom attached to the N atom was refined isotropically.

### Crystal data

**Table:**

[Fe(C_5_H_5_)(C_15_H_16_NO)]	*F*(000) = 728
*M**_r_* = 347.23	*D*_x_ = 1.417 Mg m^−^^3^
Monoclinic, *P*2_1_/*c*	Mo *K*α radiation, λ = 0.71073 Å
Hall symbol: -P 2ybc	Cell parameters from 3389 reflections
*a* = 12.1343 (4) Å	θ = 3.3–28.9°
*b* = 17.8010 (7) Å	µ = 0.93 mm^−^^1^
*c* = 7.5464 (2) Å	*T* = 293 K
β = 92.946 (3)°	Prismatic, orange
*V* = 1627.89 (9) Å^3^	0.22 × 0.18 × 0.12 mm
*Z* = 4	

### Data collection

**Table:**

Oxford Diffraction Xcalibur Sapphire3 Gemini diffractometer	3694 independent reflections
Radiation source: Enhance (Mo) X-ray Source	2843 reflections with *I* > 2σ(*I*)
Graphite monochromator	*R*_int_ = 0.029
Detector resolution: 16.3280 pixels mm^-1^	θ_max_ = 29.0°, θ_min_ = 3.3°
ω scans	*h* = −15→16
Absorption correction: multi-scan (*CrysAlis PRO*; Oxford Diffraction, 2009)'	*k* = −22→19
*T*_min_ = 0.923, *T*_max_ = 1.000	*l* = −10→9
7605 measured reflections	

### Refinement

**Table:**

Refinement on *F*^2^	Primary atom site location: structure-invariant direct methods
Least-squares matrix: full	Secondary atom site location: difference Fourier map
*R*[*F*^2^ > 2σ(*F*^2^)] = 0.039	Hydrogen site location: inferred from neighbouring sites
*wR*(*F*^2^) = 0.097	H atoms treated by a mixture of independent and constrained refinement
*S* = 1.04	*w* = 1/[σ^2^(*F*_o_^2^) + (0.0396*P*)^2^ + 0.2313*P*] where *P* = (*F*_o_^2^ + 2*F*_c_^2^)/3
3694 reflections	(Δ/σ)_max_ < 0.001
213 parameters	Δρ_max_ = 0.28 e Å^−^^3^
0 restraints	Δρ_min_ = −0.28 e Å^−^^3^

### Special details

**Table:**

Experimental. Empirical absorption correction using spherical harmonics, implemented in SCALE3 ABSPACK scaling algorithm. 'CrysAlisPro, (Oxford Diffraction, 2009)'

### Fractional atomic coordinates and isotropic or equivalent isotropic displacement parameters (Å^2^)

**Table:**

	*x*	*y*	*z*	*U*_iso_*/*U*_eq_	
Fe	0.80589 (2)	0.094395 (18)	0.51346 (4)	0.03546 (12)	
O1	0.61741 (15)	0.26845 (11)	0.5452 (2)	0.0615 (5)	
N1	0.41087 (17)	0.15206 (13)	0.4050 (2)	0.0427 (5)	
C1	0.73752 (18)	0.17593 (14)	0.6608 (3)	0.0382 (5)	
C2	0.76352 (19)	0.11125 (15)	0.7678 (3)	0.0445 (6)	
H2	0.7136	0.0826	0.8284	0.053*	
C3	0.8779 (2)	0.09863 (17)	0.7649 (3)	0.0541 (7)	
H3	0.9167	0.0604	0.8244	0.065*	
C4	0.9240 (2)	0.15390 (17)	0.6566 (3)	0.0538 (7)	
H4	0.9980	0.1580	0.6319	0.065*	
C5	0.83831 (19)	0.20199 (14)	0.5919 (3)	0.0452 (6)	
H5	0.8461	0.2432	0.5179	0.054*	
C6	0.7064 (2)	0.07479 (17)	0.2926 (3)	0.0570 (7)	
H6	0.6430	0.1015	0.2583	0.068*	
C7	0.7122 (2)	0.01088 (18)	0.4021 (3)	0.0616 (8)	
H7	0.6528	−0.0122	0.4534	0.074*	
C8	0.8221 (3)	−0.01211 (16)	0.4207 (3)	0.0595 (7)	
H8	0.8489	−0.0531	0.4860	0.071*	
C9	0.8845 (2)	0.03759 (17)	0.3236 (3)	0.0565 (7)	
H9	0.9605	0.0354	0.3135	0.068*	
C10	0.8143 (2)	0.09090 (16)	0.2445 (3)	0.0551 (7)	
H10	0.8351	0.1303	0.1724	0.066*	
C11	0.62784 (18)	0.20805 (14)	0.6183 (3)	0.0395 (5)	
C12	0.52948 (18)	0.16387 (15)	0.6742 (3)	0.0451 (6)	
H12A	0.5231	0.1704	0.8009	0.054*	
H12B	0.5430	0.1110	0.6531	0.054*	
C13	0.42057 (18)	0.18528 (15)	0.5804 (3)	0.0451 (6)	
H13A	0.3602	0.1681	0.6495	0.054*	
H13B	0.4157	0.2395	0.5705	0.054*	
C14	0.31120 (17)	0.15273 (13)	0.3047 (3)	0.0366 (5)	
C15	0.30246 (19)	0.11137 (14)	0.1457 (3)	0.0422 (6)	
C16	0.2019 (2)	0.10971 (17)	0.0517 (3)	0.0562 (7)	
H16	0.1951	0.0822	−0.0531	0.067*	
C17	0.1113 (2)	0.14773 (18)	0.1089 (3)	0.0621 (8)	
H17	0.0443	0.1455	0.0436	0.075*	
C18	0.1206 (2)	0.18863 (17)	0.2619 (3)	0.0551 (7)	
H18	0.0597	0.2144	0.3007	0.066*	
C19	0.22021 (18)	0.19193 (15)	0.3598 (3)	0.0442 (6)	
H19	0.2262	0.2206	0.4629	0.053*	
C20	0.4006 (2)	0.06922 (17)	0.0836 (3)	0.0593 (7)	
H20A	0.3801	0.0429	−0.0241	0.089*	
H20B	0.4254	0.0339	0.1731	0.089*	
H20C	0.4589	0.1040	0.0622	0.089*	
H1N	0.463 (2)	0.1487 (15)	0.361 (3)	0.052 (9)*	

### Atomic displacement parameters (Å^2^)

**Table:**

	*U*^11^	*U*^22^	*U*^33^	*U*^12^	*U*^13^	*U*^23^
Fe	0.04343 (19)	0.0331 (2)	0.02940 (16)	−0.00300 (14)	−0.00236 (12)	−0.00362 (14)
O1	0.0637 (11)	0.0499 (13)	0.0697 (12)	−0.0005 (9)	−0.0097 (9)	0.0184 (10)
N1	0.0387 (11)	0.0526 (14)	0.0366 (9)	0.0025 (10)	0.0018 (9)	−0.0108 (10)
C1	0.0475 (12)	0.0382 (14)	0.0286 (10)	−0.0034 (11)	−0.0008 (9)	−0.0081 (10)
C2	0.0543 (14)	0.0534 (17)	0.0253 (10)	0.0012 (12)	−0.0033 (9)	−0.0030 (10)
C3	0.0615 (15)	0.0616 (19)	0.0371 (12)	0.0104 (14)	−0.0174 (11)	−0.0100 (13)
C4	0.0417 (13)	0.066 (2)	0.0525 (14)	−0.0044 (13)	−0.0062 (11)	−0.0228 (14)
C5	0.0523 (13)	0.0360 (14)	0.0470 (12)	−0.0104 (11)	−0.0011 (11)	−0.0123 (11)
C6	0.0605 (16)	0.063 (2)	0.0455 (13)	0.0130 (14)	−0.0213 (12)	−0.0222 (14)
C7	0.0712 (18)	0.063 (2)	0.0509 (15)	−0.0302 (16)	0.0080 (13)	−0.0229 (15)
C8	0.092 (2)	0.0336 (15)	0.0512 (14)	0.0059 (15)	−0.0086 (14)	−0.0064 (12)
C9	0.0569 (15)	0.0592 (19)	0.0537 (14)	0.0019 (14)	0.0053 (12)	−0.0216 (14)
C10	0.0856 (19)	0.0499 (17)	0.0303 (11)	−0.0067 (15)	0.0063 (12)	−0.0028 (12)
C11	0.0498 (13)	0.0404 (14)	0.0276 (10)	−0.0020 (11)	−0.0043 (9)	−0.0072 (10)
C12	0.0511 (13)	0.0520 (16)	0.0317 (10)	−0.0039 (12)	−0.0020 (10)	−0.0023 (11)
C13	0.0446 (12)	0.0535 (16)	0.0372 (11)	0.0015 (12)	0.0017 (10)	−0.0112 (11)
C14	0.0387 (11)	0.0341 (13)	0.0369 (11)	−0.0062 (10)	0.0008 (9)	0.0024 (10)
C15	0.0497 (13)	0.0415 (15)	0.0354 (11)	−0.0109 (11)	0.0018 (10)	−0.0002 (10)
C16	0.0656 (17)	0.0599 (19)	0.0420 (12)	−0.0195 (14)	−0.0068 (12)	−0.0023 (13)
C17	0.0501 (15)	0.078 (2)	0.0561 (15)	−0.0146 (15)	−0.0152 (12)	0.0190 (15)
C18	0.0456 (14)	0.0583 (19)	0.0612 (15)	0.0025 (13)	0.0007 (12)	0.0160 (14)
C19	0.0446 (13)	0.0440 (15)	0.0437 (12)	0.0000 (11)	0.0008 (10)	0.0024 (11)
C20	0.0690 (17)	0.0640 (19)	0.0454 (13)	−0.0064 (15)	0.0067 (12)	−0.0200 (14)

### Geometric parameters (Å, º)

**Table:**

Fe—C7	2.028 (3)	C7—C8	1.395 (4)
Fe—C1	2.031 (2)	C7—H7	0.9300
Fe—C9	2.031 (2)	C8—C9	1.397 (4)
Fe—C8	2.034 (3)	C8—H8	0.9300
Fe—C2	2.034 (2)	C9—C10	1.389 (4)
Fe—C5	2.037 (2)	C9—H9	0.9300
Fe—C6	2.037 (2)	C10—H10	0.9300
Fe—C10	2.039 (2)	C11—C12	1.507 (3)
Fe—C4	2.045 (2)	C12—C13	1.515 (3)
Fe—C3	2.049 (2)	C12—H12A	0.9700
O1—C11	1.212 (3)	C12—H12B	0.9700
N1—C14	1.393 (3)	C13—H13A	0.9700
N1—C13	1.449 (3)	C13—H13B	0.9700
N1—H1N	0.74 (2)	C14—C19	1.388 (3)
C1—C5	1.431 (3)	C14—C15	1.407 (3)
C1—C2	1.432 (3)	C15—C16	1.380 (3)
C1—C11	1.469 (3)	C15—C20	1.503 (3)
C2—C3	1.408 (3)	C16—C17	1.379 (4)
C2—H2	0.9300	C16—H16	0.9300
C3—C4	1.413 (4)	C17—C18	1.365 (4)
C3—H3	0.9300	C17—H17	0.9300
C4—C5	1.414 (3)	C18—C19	1.385 (3)
C4—H4	0.9300	C18—H18	0.9300
C5—H5	0.9300	C19—H19	0.9300
C6—C7	1.406 (4)	C20—H20A	0.9600
C6—C10	1.406 (4)	C20—H20B	0.9600
C6—H6	0.9300	C20—H20C	0.9600
			
C7—Fe—C1	120.96 (11)	C1—C5—Fe	69.19 (13)
C7—Fe—C9	67.46 (11)	C4—C5—H5	126.1
C1—Fe—C9	164.08 (11)	C1—C5—H5	126.1
C7—Fe—C8	40.19 (11)	Fe—C5—H5	126.2
C1—Fe—C8	154.66 (11)	C7—C6—C10	107.3 (2)
C9—Fe—C8	40.20 (11)	C7—C6—Fe	69.41 (14)
C7—Fe—C2	109.60 (10)	C10—C6—Fe	69.88 (14)
C1—Fe—C2	41.25 (9)	C7—C6—H6	126.3
C9—Fe—C2	152.81 (11)	C10—C6—H6	126.3
C8—Fe—C2	119.71 (11)	Fe—C6—H6	126.0
C7—Fe—C5	154.91 (12)	C8—C7—C6	108.3 (2)
C1—Fe—C5	41.17 (9)	C8—C7—Fe	70.14 (15)
C9—Fe—C5	125.61 (11)	C6—C7—Fe	70.12 (15)
C8—Fe—C5	162.90 (11)	C8—C7—H7	125.8
C2—Fe—C5	69.00 (10)	C6—C7—H7	125.8
C7—Fe—C6	40.47 (11)	Fe—C7—H7	125.5
C1—Fe—C6	109.19 (10)	C7—C8—C9	107.6 (3)
C9—Fe—C6	67.52 (11)	C7—C8—Fe	69.67 (16)
C8—Fe—C6	67.82 (11)	C9—C8—Fe	69.81 (15)
C2—Fe—C6	128.99 (10)	C7—C8—H8	126.2
C5—Fe—C6	119.76 (11)	C9—C8—H8	126.2
C7—Fe—C10	67.72 (11)	Fe—C8—H8	125.9
C1—Fe—C10	127.57 (10)	C10—C9—C8	108.7 (2)
C9—Fe—C10	39.91 (11)	C10—C9—Fe	70.32 (14)
C8—Fe—C10	67.56 (11)	C8—C9—Fe	69.99 (15)
C2—Fe—C10	166.37 (11)	C10—C9—H9	125.6
C5—Fe—C10	107.41 (11)	C8—C9—H9	125.6
C6—Fe—C10	40.37 (11)	Fe—C9—H9	125.6
C7—Fe—C4	164.04 (13)	C9—C10—C6	108.0 (2)
C1—Fe—C4	68.63 (9)	C9—C10—Fe	69.76 (14)
C9—Fe—C4	106.77 (10)	C6—C10—Fe	69.75 (13)
C8—Fe—C4	125.95 (12)	C9—C10—H10	126.0
C2—Fe—C4	68.27 (10)	C6—C10—H10	126.0
C5—Fe—C4	40.53 (10)	Fe—C10—H10	126.0
C6—Fe—C4	152.92 (12)	O1—C11—C1	121.1 (2)
C10—Fe—C4	118.16 (11)	O1—C11—C12	121.6 (2)
C7—Fe—C3	127.89 (12)	C1—C11—C12	117.2 (2)
C1—Fe—C3	68.51 (10)	C11—C12—C13	115.0 (2)
C9—Fe—C3	118.55 (11)	C11—C12—H12A	108.5
C8—Fe—C3	107.98 (11)	C13—C12—H12A	108.5
C2—Fe—C3	40.32 (9)	C11—C12—H12B	108.5
C5—Fe—C3	68.25 (11)	C13—C12—H12B	108.5
C6—Fe—C3	166.04 (12)	H12A—C12—H12B	107.5
C10—Fe—C3	151.91 (12)	N1—C13—C12	110.61 (18)
C4—Fe—C3	40.37 (11)	N1—C13—H13A	109.5
C14—N1—C13	121.34 (19)	C12—C13—H13A	109.5
C14—N1—H1N	120 (2)	N1—C13—H13B	109.5
C13—N1—H1N	115 (2)	C12—C13—H13B	109.5
C5—C1—C2	107.3 (2)	H13A—C13—H13B	108.1
C5—C1—C11	125.2 (2)	C19—C14—N1	121.6 (2)
C2—C1—C11	127.4 (2)	C19—C14—C15	119.5 (2)
C5—C1—Fe	69.63 (13)	N1—C14—C15	118.9 (2)
C2—C1—Fe	69.49 (13)	C16—C15—C14	118.4 (2)
C11—C1—Fe	123.35 (14)	C16—C15—C20	121.5 (2)
C3—C2—C1	108.0 (2)	C14—C15—C20	120.1 (2)
C3—C2—Fe	70.41 (13)	C17—C16—C15	121.8 (2)
C1—C2—Fe	69.26 (11)	C17—C16—H16	119.1
C3—C2—H2	126.0	C15—C16—H16	119.1
C1—C2—H2	126.0	C18—C17—C16	119.6 (2)
Fe—C2—H2	125.9	C18—C17—H17	120.2
C2—C3—C4	108.5 (2)	C16—C17—H17	120.2
C2—C3—Fe	69.26 (12)	C17—C18—C19	120.4 (3)
C4—C3—Fe	69.64 (13)	C17—C18—H18	119.8
C2—C3—H3	125.7	C19—C18—H18	119.8
C4—C3—H3	125.7	C18—C19—C14	120.3 (2)
Fe—C3—H3	126.9	C18—C19—H19	119.8
C3—C4—C5	108.4 (2)	C14—C19—H19	119.8
C3—C4—Fe	69.99 (14)	C15—C20—H20A	109.5
C5—C4—Fe	69.44 (13)	C15—C20—H20B	109.5
C3—C4—H4	125.8	H20A—C20—H20B	109.5
C5—C4—H4	125.8	C15—C20—H20C	109.5
Fe—C4—H4	126.4	H20A—C20—H20C	109.5
C4—C5—C1	107.8 (2)	H20B—C20—H20C	109.5
C4—C5—Fe	70.03 (14)		

### Hydrogen-bond geometry (Å, º)

Cg is the centroid of the C14–C19 ring.

**Table:**

*D*—H···*A*	*D*—H	H···*A*	*D*···*A*	*D*—H···*A*
C12—H12*A*···O1^i^	0.97	2.38	3.182 (3)	139
C19—H19···*Cg*1^i^	0.93	2.98	3.838 (3)	160

Symmetry code: (i) *x*, −*y*+1/2, *z*+1/2.
